# Protective effects of dexmedetomidine in vital organ injury: crucial roles of autophagy

**DOI:** 10.1186/s11658-022-00335-7

**Published:** 2022-05-04

**Authors:** Shankun Zhao, Weizhou Wu, Xuezheng Lin, Maolei Shen, Zhenyu Yang, Sicong Yu, Yu Luo

**Affiliations:** 1 Department of Urology, Taizhou Central Hospital (Taizhou University Hospital), Taizhou, 318000 Zhejiang China; 2 Department of Urology, Maoming People’s Hospital, Maoming, 525000 Guangdong China; 3 Department of Anesthesia Surgery, Taizhou Central Hospital (Taizhou University Hospital), Taizhou, 318000 China

**Keywords:** Dexmedetomidine (DEX), Autophagy, Organ injury, Protection, Mechanism

## Abstract

Vital organ injury is one of the leading causes of global deaths. Accumulating studies have demonstrated that dexmedetomidine (DEX) has an outstanding protective effect on multiple organs for its antiinflammatory and antiapoptotic properties, while the underlying molecular mechanism is not clearly understood. Autophagy, an adaptive catabolic process, has been found to play a crucial role in the organ-protective effects of DEX. Herein, we present a first attempt to summarize all the evidence on the proposed roles of autophagy in the action of DEX protecting against vital organ injuries via a comprehensive review. We found that most of the relevant studies (17/24, 71%) demonstrated that the modulation of autophagy was inhibited under the treatment of DEX on vital organ injuries (e.g. brain, heart, kidney, and lung), but several studies suggested that the level of autophagy was dramatically increased after administration of DEX. Albeit not fully elucidated, the underlying mechanisms governing the roles of autophagy involve the antiapoptotic properties, inhibiting inflammatory response, removing damaged mitochondria, and reducing oxidative stress, which might be facilitated by the interaction with multiple associated genes (i.e., hypoxia inducible factor-1α, p62, caspase-3, heat shock 70 kDa protein, and microRNAs) and signaling cascades (i.e., mammalian target of rapamycin, nuclear factor-kappa B, and c-Jun N-terminal kinases pathway). The authors conclude that DEX hints at a promising strategy in the management of vital organ injuries, while autophagy is crucially involved in the protective effect of DEX.

## Introduction

Vital organ injury (i.e., cerebral, myocardial, renal, and lung injury) is one of the leading causes of global deaths and seriously affects the lives of patients, resulting in great healthcare and significant economic impacts in today’s society [1]. Acute organ injury occurs frequently in the perioperative period, while chronic injury is commonly caused by long-lasting stimulation and toxic insult. Ischemia–reperfusion (I/R) injury is a major cause of acute organ injury. I/R injury develops in response to interruption in the blood supply to an area of tissue, leading to persistent tissue hypoxia and severe microvascular dysfunction [2]. With the subsequent return of blood flow and oxygen supply on reperfusion, further organ injury occurs following oxidative stress and the action of proinflammatory chemokines and cytokines [3, 4]. I/R-mediated microcirculatory dysfunction can cause multiple organ injuries followed by the acute, subacute, and chronic phases after reperfusion, resulting in stepwise organ fibrosis and failure [5]. Chronic organ injury is often correlated with the rewiring of a complex metabolic network, imbalance of immune function, and tissue remodeling [6]. Acute, repeated, and chronic injuries without interventions commonly cause organ dysfunction. Consistently, intensive efforts have been made to develop novel therapeutic measures to effectively prevent or treat vital organ injuries.

Dexmedetomidine (DEX), a selective alpha_2_ adrenoceptor agonist, not only exerts sedative and anxiolytic effects but also exhibits sympathetic nerve suppression and antiinflammatory properties. Thus, it is broadly applied in clinical anesthesia and the intensive care unit (ICU) [7]. Basic and translational studies suggest that DEX is superior to some types of sedatives (i.e., benzodiazepines and propofol) in terms of multiple clinical outcomes, such as delirium, coma, subsequent infection, mechanical ventilation, and even 28-day mortality [8–10]. Accordingly, present sedation guidelines recommend DEX use over benzodiazepines for light-to-moderate sedation in critically ill patients [11]. In addition, DEX is not solely recommended for adult use as a short-term medication (< 24 h) for analgesia and sedation in the ICU but can also be applied for more than 24 h in ICU [12, 13]. Recently, mounting evidence has confirmed that DEX has an outstanding protective effect on multiple organs. Due to the antiinflammatory reaction and immunoregulation developed by DEX, numerous clinical trials support the notion that DEX confers multiorgan protection in acute organ injury events as well as during the perioperative period [14–16]. Also, mounting experimental studies have demonstrated that DEX protects against various organ injuries using different animal models [17–19], while the mechanisms underlying this protective effect are not completely understood and are currently under investigation.

A growing body of research has revealed that autophagy might be involved in the organ-protective actions of DEX [20, 21]. Autophagy, an adaptive catabolic process, functions to maintain cellular homeostasis by engulfing cellular targets, including damaged organelles, unfolded proteins, and pathogens [22–24]. Upon diverse stress conditions, the activation and inhibition of autophagy have been speculated to play roles in the protection against organ injury. Under different interventions, altered autophagy has frequently been identified in the process of treating organ injuries [25, 26]. Similarly, autophagy abnormalities are also observed under DEX treatment of vital organ injuries, including the brain [27], heart [28], kidneys [29], liver [17], and lungs [30].

Since DEX has crucial clinical implications for treating vital organs injuries, elucidating the underlying molecular mechanisms is of pivotal importance. Autophagy may be one of the key regulators in the action of DEX protecting against organ injury. However, to the best of the authors’ knowledge, there have been no comprehensive reviews on the relationship between the DEX-mediated autophagy pathway and the treatment of vital organs injuries. Therefore, it is timely to summarize and discuss the current evidence on this issue.

### Pharmacokinetic properties of DEX

4-[(1*S*)-1-(2,3-dimethylphenyl)ethyl]-1*H*-imidazole (DEX) is the dextro-enantiomer of medetomidine, with molecular formula C_13_H_16_N_2_ (molecular mass 236.7 g/mol; octanol/water partition coefficient 2.89) [31, 32]. DEX is currently approved for intravenous use, while the loading doses and infusion rates are based on a milligram per kilogram total body weight. DEX shows high protein binding (94% is bound to albumin and α1-glycoprotein) with an extensive volume of distribution (1.31–2.46 L/kg) and simply crosses the blood–brain barrier [32]. The elimination half-life of DEX in the adult health population and ICU patients is 2.1–3.1 h and 2.2–3.7 h, respectively [32, 33], while the metabolic clearance in adult patients and ICU patients is 36–42 l/h and 31.8–57 l/h, respectively [33, 34]. In children, the elimination half-life of DEX is approximate 2 h [35].

The pharmacodynamics of DEX includes sedative and hypnotic effects, analgesic effects, cardiovascular effects, respiratory effects, etc. The sedative and hypnotic effects developed by DEX may be associated with activation of central presynaptic and postsynaptic alpha_2_ adrenoceptor in the locus coeruleus, regulation of endogenous sleep-promoting pathways, and an impact on the γ-aminobutyric acid system [32]. Significant and rousable sedation effects induced by DEX are recorded at plasma concentrations between 0.2 and 0.3 ng/mL. The analgesic effects of DEX are thought to be mediated through alteration of perception and reduction of anxiety. DEX has a biphasic hemodynamic effect on the cardiovascular system, showing that low plasma concentrations induce hypotension whereas higher concentrations lead to pulmonary and systemic hypertension [36]. As reported, the hypertensive effects of DEX overcome the hypotensive effects at concentrations between 1.9 and 3.2 ng/mL [36]. Minimal respiratory depression is observed at therapeutic plasma concentrations up to 2.4 ng/mL, showing a preservation of ventilatory response to CO_2_ [37]. The ventilatory frequency can elevate with dose escalation of DEX, which compensates for slightly decreased tidal volumes [32]. With target concentrations between 0.2 and 0.6 ng/mL of DEX, no relevant pharmacokinetic interactions were identified in DEX when combining with propofol, isoflurane, midazolam, or alfentanil [32].

### Current knowledge

α2-Receptors are frequently detected in various vital organs, including the central nervous system, kidneys, lungs, and liver [32]. Since DEX is a highly selective α2 adrenoceptor agonist, it may mediate a broad spectrum of pharmacodynamic actions on these organs. In numerous animal studies [18, 38, 39], DEX appears to alleviate the inflammation responses and the I/R injury of multiple organs, i.e., the brain, liver, and intestines. More importantly, although α2 adrenoceptor is not found in the myocardium, a large body of previous studies suggest that DEX plays a protective role on myocardial I/R injury [40, 41]. DEX-mediated modulation of autophagy is considered to play the adrenergic receptor agonist’s protective role in multiple organ injuries. Based on the above evidence, DEX exerts an encouraging protective effect on multiple organs. Mechanistically, recent experimental research has suggested that autophagy might be involved in this action. In this review, we thus outline the molecular and biological functions of autophagy in DEX-mediated organ-protective effects.

### Literature search

A comprehensive review of the literature was undertaken using six databases (MEDLINE, EMBASE, Google Scholar, Cochrane Library, Web of Science, and PsychINFO) to identify relevant studies. The searching strategy in MEDLINE using MeSH and keywords was: (((((((((("Autophagy"[Mesh]) OR (Autophagy, Cellular)) OR (Cellular Autophagy)) OR (Autophagocytosis)) OR (Reticulophagy)) OR (ER-Phagy)) OR (ER Phagy)) OR (Nucleophagy)) OR (Ribophagy)) OR (Lipophagy)) AND ((((((("Dexmedetomidine"[Mesh]) OR (MPV-1440)) OR (MPV 1440)) OR (MPV1440)) OR (Precedex)) OR (Dexmedetomidine Hydrochloride)) OR (Hydrochloride, Dexmedetomidine)). The reference list was also reviewed to detect additional studies. A data collection table was applied to extract the key data from the relevant studies, including the first author’s name, publication year, geographical distribution, cell/animal model, types of organ injury, DEX administration, autophagy status, associated genes or pathways, and the main findings of the included studies. Finally, 24 studies [21, 27–30, 41–59] were included. Among these, 14, 4, 3, and 3 eligible studies reported cerebral injury, myocardial injury, kidney injury, and lung injury, respectively.

### Organ-protective properties of DEX and the roles of autophagy

#### Cerebral injury

Fourteen publications reported autophagy involving the action of the protective effect of DEX in brain injury. The experimental models among these eligible studies included rat, mouse, and neurocyte (i.e., astrocytes, PC12, and neuroblastoma cells). The types of central nervous injury included cerebral ischemia/reperfusion injury, traumatic brain injury, neurological injury, cognitive impairment, hippocampus injury, oxygen–glucose deprivation–reoxygenation injury, and neonatal hypoxic ischemia. The route for DEX administration in an animal model included intraperitoneal injection and intravenous injection via the femoral vein or the caudal vein. The dose of DEX in a rat model ranged from 3 to 50 μg/kg, but 20–25 μg/kg in a mouse model. DEX in a cell model was administrated through cell supernatants, while the dose of DEX was 1 μM. Most of the included studies (12/14, 86%) reported the status of autophagy was inhibition in the protective effect of DEX in cerebral injury. Multiple genes and signaling pathways have been found to be involved in autophagy-mediated neuroprotection by DEX.

**Fig. 1:**
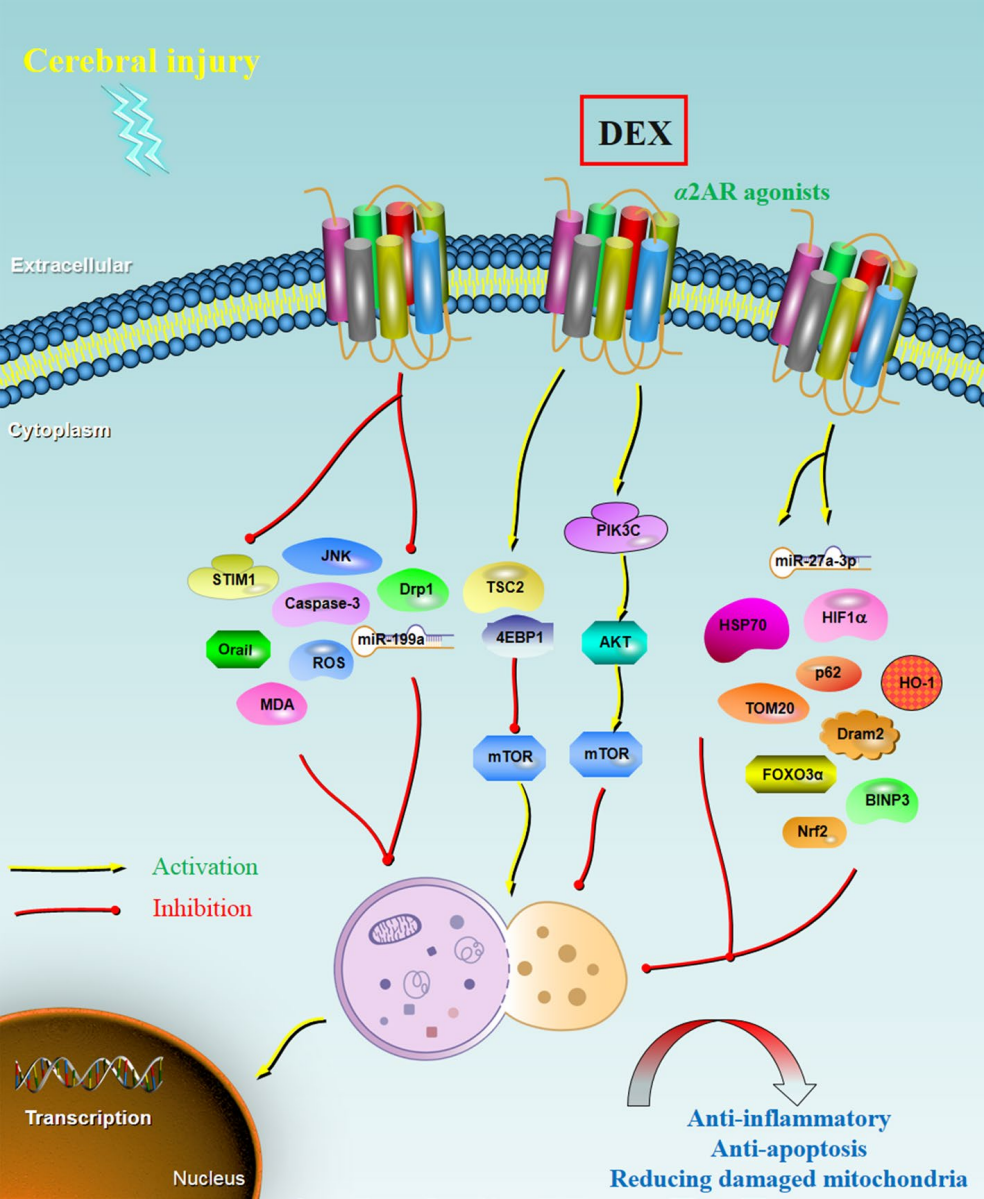
Main mechanisms of autophagy in the cerebra-protective effects of DEX. DEX is one the α2AR agonists. Under administration of DEX in treating cerebral injury, the autophagy level was regulated by multiple associated genes and a series of downstream signaling, resulting in reduction of inflammatory, apoptosis, and damaged mitochondria. *DEX* dexmedetomidine, *HIF-1α* hypoxia inducible factor-1α, *LC3* light chain 3 B, *Drp1* dynamin-related protein 1, *HSP70* heat shock 70 kDa protein, *TOM20* translocase of outer mitochondrial membrane 20, *Dram2* DNA damage regulated autophagy modulator 2, *FOXO3α* forkhead-box-protein 3α, *TSC2* tuberous sclerosis complex 2, *STIM1* stromal interaction molecule 1, *ROS* reactive oxygen species, *MDA* malondialdehyde, *Nrf2* nuclear factor erythroid 2-related factor 2

The characteristics and the main findings of the 14 relevant studies reporting cerebral injury are summarized in Table 1. Figure 1 shows the main mechanisms of autophagy in the cerebra-protective effects of DEX.

**Table 1:** Characteristics and main findings of relevant studies reporting on cerebral injury

Study/ref.	Experimental model	Types of injury	DEX administration	Status of autophagy	Associated genes or pathways	Main findings
Luo et al. 2017 [27]	Mouse	Ischemic cerebral injury	Intraperitoneally, 25 μg/kg	Inhibited	Upregulating HIF-1α	Postconditioning with DEX at beginning of reperfusion protects mouse brain from ischemia–reperfusion injury via inhibition of neuronal autophagy by upregulating of Bcl-1, p62, and HIF-1α and downregulating of LC3 and Beclin 1
Shen et al. 2017 [42]	Rat	Traumatic brain injury	Injected via the left femoral vein, 15 μg/kg	Inhibited	Activation of the PI3K/Akt/ mTOR pathway	DEX alleviates the degree of traumatic brain injury via inhibition of neuronic autophagy by activating PI3K/AKT/mTOR signaling pathway
Shan et al. 2018 [43]	Pregnant rat	Neurological injury	Intraperitoneally, 20 μg/kg	Inhibited	Upregulating Bcl2, downregulating Drp1 and Bax	DEX improved the abnormal morphology of hippocampal CA1 regions of rat-pup brains and inhibited sevoflurane-induced activation of autophagy
Yi et al. 2018 [44]	Rat	Cognitive impairment	Intraperitoneally, 4 μg/kg	Inhibited	Downregulating of LC3-I, LC3-II, and Beclin-1	DEX improved the cognitive dysfunction in aged rats under sevoflurane anesthesia by decreasing autophagy of hippocampal neurons
Lu et al. 2019 [45]	Rat	Cerebral ischemia‑reperfusion injury	Intraperitoneally, 50 μg/kg	Inhibited	Downregulation of Bax and Caspase-3, upregulation in Bcl‑2 and HSP70	DEX exerts neuroprotective effect by inducing mild hypothermia, slowing down heart rate, attenuating apoptosis of neurocytes, and repressing autophagy
Zhu et al. 2019 [46]	Rat	Cerebral ischemia/reperfusion injury	Left femoral vein, 3 μg/kg	Inhibited	Inhibiting the activation of JNK signaling pathway	The effect of DEX might be related to the inhibition of JNK pathway activation, and to affect the expressions of inflammatory factors and autophagy-related proteins
Tang et al. 2019 [47]	SH-SY5Y cells	Ischemia/reperfusion cerebral injury	Treated with 1 μM DEX for 18 h reoxygenation	Inhibited	Downregulating LC3 and Beclin 1, upregulating BCL-2, p62, and TOM20	DEX increased the cell survival meanwhile reduced the production of autophagic vesicles, as well as regulated some related proteins
Li et al. 2020 [50]	Rat	Traumatic brain injury	Intraperitoneally, 20 μg/kg	Inhibited	Inactivation of the circLrp1b/miR-27a-3p/Dram2 signaling pathway	DEX inhibits inflammatory response and autophagy in a traumatic brain injury rat model by acting on the circLrp1b/miR-27a-3p/Dram2 pathway
Yu et al. 2020 [21]	Rat	Hippocampus injury	Intraperitoneally, 25 μg/kg	Activated mitophagy	Increased expression levels of FOXO3α, BINP3, LC3-II/LC3-I, and P62	DEX attenuated hippocampus injury and improved cognitive function by activating SIRT3-mediated mitophagy and inhibiting activation of the NLRP3 inflammasome
Zhu et al. 2020 [48]	Mouse and astrocytes	Cerebral ischemia	1 μM, via medium	Activated astrocytes autophagy	Upregulating TSC2 and 4EBP1, downregulating mTOR	DEX increases the viability and inhibits apoptosis of astrocytes exposed to oxygen–glucose deprivation, which might be related to the activation of autophagy by regulating TSC2/mTOR pathway
Hu et al. 2020 [49]	PC12 Cells	OGD/R injury	1 μg/mL, via medium	Inhibited	Decreasing the levels of STIM1 and Orai1 proteins	DEX attenuates cell apoptosis following OGD/R by inhibiting autophagy in PC12 cells, which may be correlated to the repression of Ca^2+^-STIM1/Orai1 signaling
Zhu et al. 2021 [52]	Rat	Cerebral ischemia/reperfusion	Caudal vein, 3 µg/kg/h	Inhibited	Inhibiting the expression of miR‑199a	DEX inhibited autophagy and decreased nerve cell injury by decreasing the level of miR‑199a
Xue et al. 2021 [51]	Rat	Neonatal hypoxic ischemia	Intraperitoneally, 25 μg/kg	Inhibited	Down-regulating LC3B-II and Beclin 1	Protective effects of DEX were evidenced by the inhibition of excessive autophagy of neurons and microglia, reducing the decline of long-term neuronal density and axon demyelination
Feng et al. 2021 [53]	Mouse	Traumatic brain injury	Intraperitoneally, 20 μg/kg	Inhibited	Decreasing the levels of ROS and MDA, and increasing the expression of Nrf2 and HO‑1	DEX improves neurological outcomes and reduces neuronal death by protecting against neural autophagy and neuroinflammation by regulating the ROS/Nrf2 pathway

*OGD/R* oxygen–glucose deprivation–reoxygenation

### mTOR signaling pathway

The phosphoinositide 3-kinase (PI3K)/Akt/mammalian target of rapamycin (mTOR) pathway is one of the most important signaling pathways with a critical biological function in various diseases [60, 61], including neurological disorders [62]. As reported, numerous drugs exert their neuroprotective effect via the PI3K/Akt/mTOR signaling pathway [63]. mTOR is considered to serve as a central player in the regulation of autophagy because it can inhibit autophagy in the process of growth factors and abundant nutrients [64]. Shen et al. [42] demonstrated that DEX alleviated the degree of traumatic brain injury via inhibition of neuronic autophagy by activating the PI3K/AKT/mTOR signaling pathway. In line with this finding, some investigators also found that the inhibition of neuronic autophagy was one of the therapeutic targets for traumatic brain injury treatment [65]. Also, the protective effects of DEX are speculated to be against the process of autophagy and apoptosis. Zhu et al. [48] reported that DEX increased the viability and inhibits apoptosis of astrocytes exposed to oxygen–glucose deprivation, which might be related to the activation of autophagy by regulating the tuberous sclerosis complex 2 (TSC2)/mTOR pathway. The authors indicated that DEX treatment could upregulate the expression of TSC2 and subsequently reduce the phosphorylation of mTOR. In contrast to Shen et al.’s study, Zhu et al. found that the protective effect played by DEX might be associated with augmented autophagy of astrocytes. Consistent with Zhu et al.’s findings, Yu et al. [21] demonstrated that DEX attenuated hippocampus injury by activating SIRT3-mediated mitophagy. Of note, Zhu et al. and Yu, et al.’s studies are the only two included studies (2/14, 14%) reporting that the status of autophagy is activation when treating with DEX for cerebral injury. Commonly, autophagy is activated in cerebral injury [66], while DEX may inhibit the autophagy level and thus contribute to the neuroprotection in cerebral damage [53]. With the same cell line of astrocytes as used in Zhu et al.’s study [48], Qin et al. [67] suggested that the inhibition of autophagy might exert the protective effect on astrocytes after ischemic astrocyte injury. This could be partially explained by the finding that autophagy may play different roles in different cerebral injury stages, i.e., ischemia and reperfusion [48]. The exact roles of autophagy at different timepoints after cerebral injury deserve further investigation.

### MicroRNAs (miRNAs)

miRNAs are a major class of conserved short noncoding RNAs with crucial biological functions in the regulation of a third of the whole genome at the posttranslational level [68]. miRNAs exert their roles by increasing messenger RNA degradation or by blocking messenger RNA translation [69]. Numerous studies have suggested that there is a close association between miRNAs and autophagy in various diseases, including cerebral injury [70]. Also, miRNAs-mediated autophagy and the signaling cascades might play critical roles in the effect of DEX in protecting cerebral injury. Li et al. [50] showed that DEX could improve the neurological outcome in a traumatic brain injury rat model by inhibiting autophagy and regulating the circLrp1b/miR-27a-3p/Dram2 pathway. They found that the protective effect of DEX after cerebral injury might be attributable to the downregulation of circLrp1b and the inhibition of injury-induced autophagy, while these effects were dramatically abolished by miR-27a-3p suppression. Zhu et al. reported that the autophagy level in the cerebral cortex increased in an animal model of cerebral ischemia/reperfusion injury, while inhibited autophagy was observed after treating with DEX. During this action, Zhu et al. further found that DEX significantly inhibited the expression of miR‑199a and thus improved neurocyte injury. The above evidence indicated that inhibition of autophagy might be involved in the DEX-induced neuroprotective effect in cerebral injury.

### Autophagy-associated proteins (Beclin-1, Bcl-2, LC3-I, and LC3-II)

The therapeutic implications of DEX in brain injury may also be strongly associated with the altered expression of autophagy-associated proteins such as Beclin-1, Bcl-2, LC3-I, and LC3-II. Beclin-1 is involved in the initiation and maturation steps of autophagy, constituting the primary component of the autophagy mechanism [71]. Bcl-2, one of the key interacting proteins of Beclin-1 and the antiapoptotic family members, can suppress autophagy initiation by inhibiting the cascade of autophagy formation [72]. Both LC3-I and LC3-II are biomarkers for autophagy. LC3B-II/I indicates the generation of autophagosomes. Shan et al. [43] found that DEX improved the abnormal morphology of hippocampal CA1 regions of rat-pup brains by inhibiting sevoflurane-induced activation of autophagy via upregulating Bcl-2. Lu et al. demonstrated that DEX exerted a neuroprotective effect by repressing autophagy in a cerebral ischemia/reperfusion injury rat model, which was partially caused by the upregulation of Bcl‑2 expression. Xue et al. [51] showed that the protective effects of DEX were evidenced by the inhibition of excessive autophagy of neurons and microglia through downregulating LC3B-II and Beclin1. In line with Xue et al.’s findings, Yi et al. [44] found that the protective functioning developed by DEX might be via decreasing autophagy of hippocampal neurons, which presented with the reduction of LC3-I, LC3-II, and Beclin-1 expression. In contrast, though Yu et al. [21] detected that DEX attenuated hippocampus injury, they observed that the status of mitophagy was activated, characterized by enhancing LC3-II/LC3-I expression.

### Other associated genes

In addition to the above factors, the roles of autophagy in the neuroprotective effects mediated by DEX might also be caused by some other associated proteins and signaling pathways, e.g., HIF-1α, p62, Drp1, Caspase-3, HSP70, TOM20, Dram2, FOXO3α, BINP3, TSC2, 4EBP1, STIM1, Orai1, ROS, MDA, Nrf2, HO‑1, and JNK signaling [21, 27, 43, 45–50, 53]. Among these genes, a positive correlation has been found between autophagy and Drp1, Caspase-3, Dram2, FOXO3α, BINP3, TSC2, 4EBP1, STIM1, Orai1, ROS, MDA, and JNK signaling pathway. In contrast, autophagy has a negative relationship with the expression of HIF-1α, p62, HSP70, TOM20, Nrf2, and HO‑1. All these genes and signaling cascades might be linked to biogenesis and biological functions of autophagy in the neuroprotective action of DEX.

### Myocardial injury

DEX has also been reported to elicit cardioprotective effects via various molecule mechanisms. Autophagy regulation is considered to be one of the proposed mechanisms, which is believed to constitute a crucial process in the self-preservation of the heart. As reported, autophagy machinery involves immunity modulation through transmitting microbes to lysosomes for degradation and facilitating the release of cytokines for microbe digestion [73]. To date, four experimental studies have confirmed the essential roles of autophagy in DEX-mediated cardioprotection [28, 41, 54, 55] (Table 2). According to Yu et al.’s study, DEX could attenuate septic myocardial injury by increasing autophagic flux via activating α7nAChR and the Akt/GSK-3β cascades, resulting in a reduction of the myocardium apoptosis and inflammatory response. In line with this finding, Xiao et al. also found that autophagy upregulation was associated with the action that DEX protected human cardiomyocytes against I/R injury. They further observed that α2-adrenergic receptor/AMPK signaling cascades greatly contributed to the activation of autophagy during the protective process developed by DEX.

**Table 2:** Characteristics and main findings of relevant studies reporting on myocardial, kidney, and lung injury

Study/ref.	Experimental model	Types of injury	DEX administration	Status of autophagy	Associated genes or pathways	Main findings
*Myocardial injury*						
Yu et al. 2019 [28]	Rat	Septic myocardial dysfunction	Injection, 50 μg/kg	Promoted	Upregulating 7nAChR and the PI3K/Akt pathway	DEX attenuates the myocardium injury by mediating autophagic flux; DEX decreases the myocardium apoptosis and inflammatory response mediated by increased autophagy by activating α7nAChR and the PI3K/Akt pathway
Zhang et al. 2020 [54]	Rat	Myocardial ischemia/reperfusion injury	Intravenous injection, 10 μg/kg	Inhibited	Upregulating the SIRT1/mTOR pathway	DEX reduces cardiomyocyte apoptosis, oxidative stress, and inflammatory reactions via upregulating the SIRT1/mTOR axis and decreasing overautophagy in myocardial ischemia/reperfusion injury rats
Xiao et al. 2021 [55]	Cardiomyocytes	Myocardial ischemia/reperfusion injury	5 μM DEX was added to the culture media	Upregulated	Upregulating AMP-activated protein kinase (AMPK) and phospho AMPK	DEX protected human cardiomyocytes from apoptosis and was associated with autophagy; the protection of DEX for H/R injury was AMPK dependent and α2-adrenergic receptor dependent
Li et al. 2021 [41]	Rat	Myocardial ischemia/reperfusion injury	Injected through the jugular vein catheter, 10 μg/kg	Inhibited	Upregulating Beclin1 and activating the PI3K/Akt pathway	DEX upregulates the phosphorylation of Beclin 1 at S295 site by activating the PI3K/Akt pathway and reduces the interactions of Atg14L–Beclin1–Vps34 complex, thus inhibiting autophagy and protecting against myocardial ischemia/reperfusion injury
*Kidney injury*						
Lempiainen et al. 2014 [29]	Rat	Cerebral kidney ischemia–reperfusion injury	Intravenous injection, 10 μg/kg	Enhanced	Upregulating renal p38 MAPK	DEX preconditioning ameliorates kidney ischemia–reperfusion injury and inflammatory response via the enhancement of autophagy and the regulation of the p38-CD44 pathway
Yang et al. 2020 [56]	Rat	Lipopolysaccharide-induced acute kidney injury	Intraperitoneally, 30 μg/kg	Enhanced	Upregulating the expression of p-AMPK and downregulating p-mTOR	DEX ameliorates inflammatory response by reducing NLRP3 inflammasome and inflammatory cytokines by enhancing autophagy via the AMPK/mTOR pathway
Zhao et al. 2020 [57]	Rat	Lipopolysaccharide-induced acute kidney injury	Intraperitoneally, 30 μg/kg	Enhanced	Inhibition of the PI3K/AKT/mTOR pathway	DEX protects against LPS-induced acute kidney injury by enhancing autophagy, thus removing the damaged mitochondria and reducing oxidative stress and apoptosis through the α_2_-AR and inhibition of the PI3K/AKT/mTOR pathway
*Lung injury*						
Zhang et al. 2017 [30]	Rat	Lung ischemia/reperfusion injury	Administered intravenously, 10 μg/kg	Inhibited	Upregulating the level of HIF‑1α, downregulating BNIP3, BNIP3 L, and LC3II	Preconditioning with DEX provided protection against lung injury in a dose-dependent manner by inhibiting autophagy, which might be associated with the upregulation of HIF-1α and downregulation of BNIP3 and BNIP3 L
Ding et al. 2018 [58]	Mouse	Lipopolysaccharide-induced acute lung injury	Intravenously injected, 50 μg/kg	Inhibited	Inhibition of the TLR4-NF-κB pathway	DEX protects against acute lung injury via reducing the inflammatory response and inhibiting autophagy-related proteins and signaling pathway
Li et al. 2021 [59]	Rat	Toxic shock-induced lung injury	Intraperitoneally, 50 μg/kg	Inhibited	Decreasing the expression of pERK1/2 protein	DEX protects against lung injury by inhibiting autophagy and inflammation

*iPSC cell* human induced pluripotent stem cell

Conversely, although two other studies [41, 54] have also reported that DEX treatment significantly attenuated myocardium injury, the researchers found that the autophagy status was inhibited in this process. Zhang et al. [54] demonstrated that DEX alleviated myocardial ischemia/reperfusion injury by dramatically decreasing overautophagy and reducing cardiomyocyte apoptosis, oxidative stress, and inflammatory reactions via upregulating the SIRT1/mTOR axis. DEX postconditioning could result in a decrease of LC3II and Beclin-1 and an elevation of p62 protein level, thus inhibiting autophagy. Li et al. [41] reported that DEX upregulated the phosphorylation of Beclin1 by activating the PI3K/Akt pathway and reduced the interactions of Atg14L–Beclin 1–Vps34 complex, thus inhibiting autophagy and protecting against myocardial ischemia/reperfusion injury. As shown in Table 2, DEX administration in both Zhang et al. and Li et al.’s study was based on intravenous injection, and the dose was the same at 10 μg/kg.

**Fig. 2:**
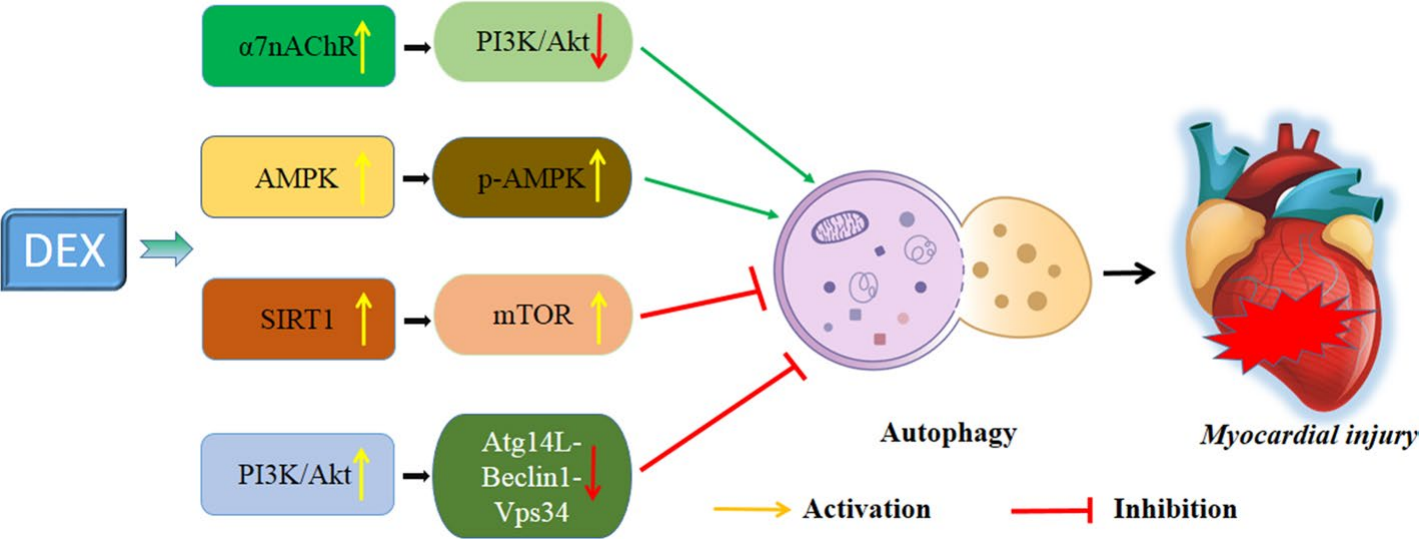
Mechanisms of autophagy in myocardial-protective effects of DEX. *7nAChR* α7 nicotinic acetylcholine receptor, *SIRT1* Sirtuin 1, *AMPK* adenosine monophosphate-activated protein kinase, *VPS34* vacuolar protein sorting 34

The mechanisms of autophagy in the myocardial-protective effects of DEX are illustrated in Fig. 2.

### Kidney injury

**Fig. 3:**
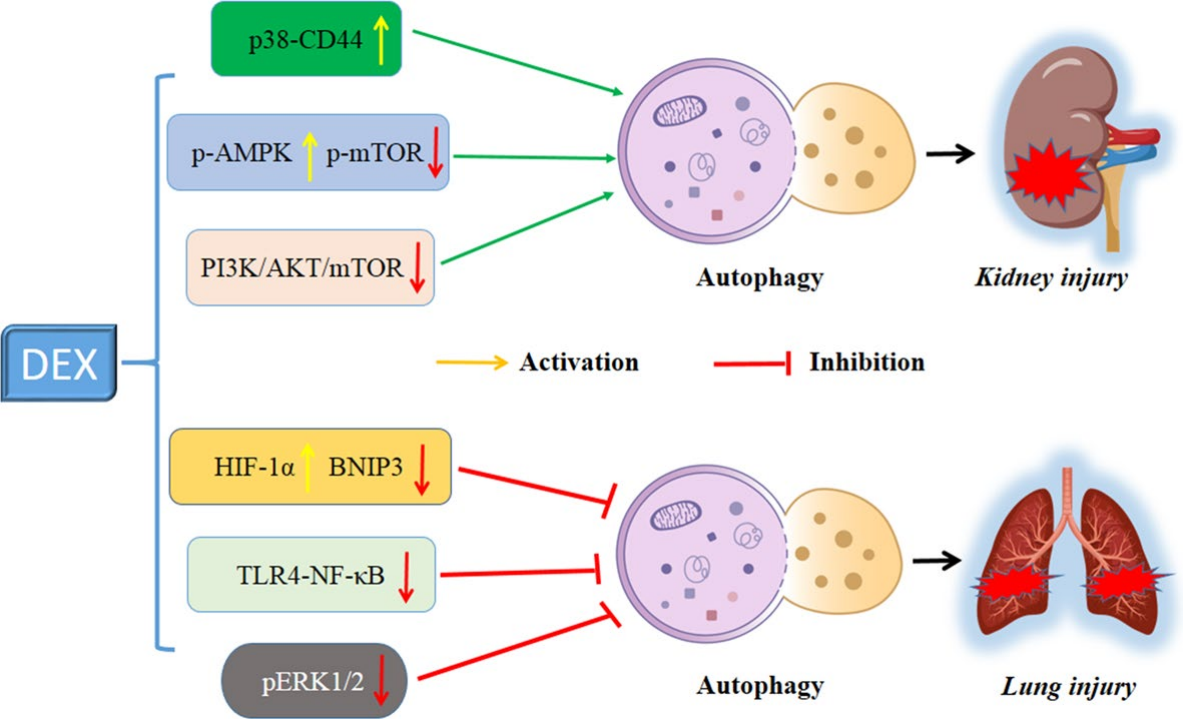
Mechanisms of autophagy in kidney- and lung-protective effects of DEX. *MAPK* mitogen-activated protein kinase, *AMPK* adenosine monophosphate-activated protein kinase, *HIF-1α* hypoxia inducible factor-1α, *BNIP3* B cell lymphoma 2 interacting protein 3, *TLR4* toll-like receptor 4, *ERK* extracellular signal regulated kinases

Acute kidney injury, a disease with high morbidity and mortality, is closely associated with multiple organ dysfunction. Kidney I/R injury and sepsis are the two main causes for the development of acute kidney injury. Autophagy has been shown to elicit some protective effects in the pathological processes of renal tubular injury [74]. A previous study [29] suggested that DEX preconditioning ameliorated kidney I/R injury and inflammatory response via the enhancement of autophagy and upregulation of the renal p38-CD44 pathway. The authors found that autophagy was markedly downregulated by kidney I/R injury, while intravenous treatment with 10 μg/kg DEX effectively prevented the impairment of the autophagic response, thus maintaining the degradation and recycling of multiple cellular components [29]. Consistent with this finding, two subsequent studies also observed that the renoprotective effects of DEX were mediated by the enhancement of autophagy after kidney injury. In a lipopolysaccharide-induced acute kidney injury rat model, Yang et al. demonstrated that DEX ameliorated the inflammatory response by reducing the NLRP3 inflammasome and inflammatory cytokines by enhancing autophagy via the AMPK/mTOR pathway. With the same acute kidney injury model, Zhao et al. found that DEX could protect against kidney injury by enhancing autophagy, thus removing damaged mitochondria and reducing oxidative stress and apoptosis through α2-AR and inhibition of the PI3K/AKT/mTOR pathway. Both animal models in Yang et al. and Zhao et al.’s studies were treated with DEX by intravenous injection with a dose of 30 μg/kg. The characteristics of the above relevant studies reporting kidney injury are summarized in Table 2. The mechanisms of autophagy in the kidney-protective effects of DEX are illustrated in Fig. 3 (upper).

### Lung injury

Acute lung injury, one of the serious forms of diffuse lung disease, has high morbidity and mortality and imposes a substantial health burden globally [75]. The common causes of acute lung injury include serious infection, burns, trauma, and shock. Fluid conservative strategy and lung-protective ventilation are the two certain supportive treatments to treat acute lung injury effectively. Recently, DEX has been suggested to exert protective effects on pulmonary functions in acute lung injury and ventilator-induced lung injury [76]. Mechanistically, the lung-protective effects developed by DEX might be correlated to the autophagy-associated signaling pathways. To date, three studies [30, 58, 59] have reported the roles of autophagy in the action of DEX attenuating lung injury. All these studies indicated that the autophagic response was inhibited when treated with DEX in an animal model of lung injury. Zhang et al. [30] reported that preconditioning with DEX protected against lung injury in a dose-dependent manner by inhibiting autophagy, which might be associated with the upregulation of HIF-1α and downregulation of BNIP3 and BNIP3 L in a lung ischemia/reperfusion injury rat model. Ding et al. [58] showed that DEX protected against lipopolysaccharide-induced acute lung injury via reducing the inflammatory response and inhibiting autophagy-related proteins and the TLR4-NF-κB signaling pathway. Based on a toxic shock-induced lung injury rat model, Li et al. [59] found that DEX remarkably protected against lung injury by inhibiting autophagy and inflammation by decreasing the expression of pERK1/2 protein. The administration of DEX was the same in the above studies, viz. 50 μg/kg DEX intravenously. The characteristics of the relevant studies reporting lung injury are listed in Table 2, while the underlying mechanisms of autophagy in the protective effects of DEX are shown in Fig. 3 (lower).

### Limitations and perspectives

To the best of the authors’ knowledge, this is the first comprehensive review to summarize all the evidence of the crucial roles of autophagy in the action of DEX protecting against vital organ injuries. First, all the included studies listed in Tables 1 and 2 were either in vivo or in vitro experiments. The exact roles of autophagy in the human body under DEX treatment in organ injury have not been fully understood yet, which deserves further investigation. Second, the level of autophagy flux in the process of the DEX-mediated protective effect on organ injury is still controversial among different studies. Most of the included studies (17/24, 71%) demonstrated that the modulation of autophagy was inhibited during this process, but the remaining studies indicated that the autophagy level was promoted. This phenomenon is particularly observed in myocardial injury, with half of the included studies reporting inhibition and half reporting enhancement of the autophagy level. This inconsistency regarding the autophagy level might be due to the various timepoints monitored in different studies. Besides, autophagy may play a dual role in the protective effect against organ injury, which needs further investigation.

## Conclusions

This review highlights the crucial roles of autophagy in the protective effect of DEX on multiple vital organs, including cerebral, myocardial, kidney, and lung injuries. The vast majority of the included studies have shown that the autophagy level is suppressed under treatment with DEX in organ injuries, but several studies suggested that the level of autophagy was dramatically increased after administration of DEX. Albeit not fully elucidated, the underlying mechanisms governing the roles of autophagy involve the antiapoptotic properties, inhibiting inflammatory response, removing damaged mitochondria, and reducing oxidative stress, which may be facilitated by the interaction with multiple associated proteins and signaling cascades. With the progress of extensive in-depth studies, DEX-mediated autophagy will be fully understood to guide better clinical applications for organ protection.

## Data Availability

Not applicable.

## Abbreviations

DEX: Dexmedetomidine
HIF-1α: Hypoxia inducible factor-1α
LC3: Light chain 3 B
Drp1: Dynamin-related protein 1
HSP70: Heat shock 70 kDa protein
TOM20: Translocase of outer mitochondrial membrane 20
Dram2: DNA damage regulated autophagy modulator 2
FOXO3α: Forkhead-box-protein 3α
TSC2: Tuberous sclerosis complex 2
STIM1: Stromal interaction molecule 1
ROS: Reactive oxygen species
MDA: Malondialdehyde
Nrf2: Nuclear factor erythroid 2-related factor 2
7nAChR: α7 nicotinic acetylcholine receptor
SIRT1: Sirtuin 1
AMPK: Adenosine monophosphate-activated protein kinase
VPS34: Vacuolar protein sorting 34
MAPK: Mitogen-activated protein kinase
BNIP3: B cell lymphoma 2 interacting protein 3
TLR4: Toll-like receptor 4
ERK: Extracellular signal regulated kinases
